# Short- and Long-Term Neurological and Psychiatric Sequelae of Developmental Exposure to Antiepileptic and Anesthetic Drugs

**DOI:** 10.3389/fneur.2015.00041

**Published:** 2015-03-05

**Authors:** Patrick A. Forcelli

**Affiliations:** ^1^Department of Pharmacology and Physiology, Georgetown University, Washington, DC, USA

**Keywords:** epilepsy, anesthesia, toxicology, development, translational research, clinical research

Phenobarbital has been in clinical use for over a century; phenytoin for more than three-quarters of a century, and volatile anesthetics, such as isoflurane and halothane for more than half a century. Despite the long history of use of these compounds for the treatment of epilepsy, and for anesthesia, their effects on brain development are still relatively poorly understood. For this reason, the use of neuroactive drugs during gestation and infancy remains one of the thorniest issues in clinical neurology and neonatology.

I had the privilege of co-editing this research topic with my mentor, colleague, and friend, Dr. Karen Gale. Dr. Gale passed away in August of 2014. She and I saw this research topic as an opportunity to highlight the state of the field, and in particular, underscore important future research directions. Together, these articles do exactly that.

In this research topic, the current state of the field is reviewed from pre-clinical and clinical perspectives by Turski and Ikonomidou (1) and Gedzelman and Meador (2). These reviews highlight converging results from animal and human studies that underscore the profound impact of these drugs can have on the developing brain. Complementing these reviews, Rochelle Caplan’s translational review focuses on the role of antiepileptic drugs in psychopathology seen in pediatric epilepsy (3). From a translational perspective, the review by Khanna and colleagues (4), and the opinion piece by Pressler and Auvin (5) highlight the importance of neuronal chloride homeostasis as a key regulator of neuronal activity during brain development and as a critical mediator of the efficacy of the first-line therapies for neonatal seizures (i.e., phenobarbital).

With respect to anesthesia, the review from Jevtovic-Todorovic and colleagues (6) approaches the problem of anesthesia-induced damage to the developing brain from a highly mechanistic perspective. In particular, they highlight the role of sub cellular organelles, such as mitochondia, in the pathophysiology of neonatal anesthesia exposure. Furthermore, the review by Cheng Wang outlines research approaches and models that have been employed in pre-clinical pediatric neurotoxicology experiments (7). Finally, the research article by Murphy and Baxter highlights sex-dependent effects of neonatal anesthesia exposure on long-term cognitive outcomes (8).

In the decade and a half since the first characterization of apoptotic neurodegeneration in the developing rat brain after exposure to these drugs (9–11), this field has made considerable advances in understanding mechanisms of toxicity and characterizing the types of lasting injury that occur after early drug exposure.

Indeed, the importance of this research was highlighted in the 2014 NINDS Benchmarks for Epilepsy Research, which lists as a goal, “[To] identify the impact of pharmacological treatment of the epilepsies on fetal and neonatal development. Develop strategies to control seizures in pregnancy without causing harm to either the mother or child” (12).

There are, however, many questions that remain to be addressed.

1. To what degree do the mechanisms of developmental neurotoxicity overlap and differ between classes of drugs (e.g., anesthesia vs. antiepileptic drugs).
2. How can pre-clinical research influence clinical practice with respect to these drugs?
3. What therapeutic strategies and adjunct treatments can be used to minimize/avoid developmental neurotoxicity?
4. How does sex modulate risk for developmental neurotoxicity?
5. What features and types of toxicity will be revealed by studies in non-human primate models?

It is my sincere hope that 10 years from now, we will have made considerable progress in addressing these issues.

## Conflict of Interest Statement

The author declares that the research was conducted in the absence of any commercial or financial relationships that could be construed as a potential conflict of interest.
